# Regional Regression Correlation Model of Microplastic Water Pollution Control Using Circular Economy Tools

**DOI:** 10.3390/ijerph20054014

**Published:** 2023-02-23

**Authors:** Valentin Marian Antohi, Romeo Victor Ionescu, Monica Laura Zlati, Catalina Iticescu, Puiu Lucian Georgescu, Madalina Calmuc

**Affiliations:** 1 Department of Business Administration, Dunarea de Jos University of Galati, 800001 Galati, Romania; 2 Department of Finance, Accounting and Economic Theory, Transylvania University of Brasov, 500036 Brasov, Romania; 3 Department of Administrative Sciences and Regional Studies, Dunarea de Jos University of Galati, 800201 Galati, Romania; 4 Department of Chemistry, Physics and Environment, REXDAN Research Infrastructure, Dunarea de Jos University of Galati, 800008 Galati, Romania; 5 REXDAN Research Infrastructure, Dunarea de Jos University of Galati, 800008 Galati, Romania

**Keywords:** microplastics, water pollution, water anti-pollution policies, circular economy

## Abstract

Water pollution caused by microplastics represents an important challenge for the environment and people’s health. The weak international regulations and standards in this domain support increased water pollution with microplastics. The literature is unsuccessful in establishing a common approach regarding this subject. The main objective of this research is to develop a new approach to necessary policies and ways of action to decrease water pollution caused by microplastics. In this context, we quantified the impact of European water pollution caused by microplastics in the circular economy. The main research methods used in the paper are meta-analysis, statistical analysis and an econometric approach. A new econometric model is developed in order to assist the decision makers in increasing efficiency of public policies regarding water pollution elimination. The main result of this study relies on combining, in an integrated way, the Organisation for Economic Co-operation and Development’s (OECD) data on microplastic water pollution and identifying relevant policies to combat this type of pollution.

## 1. Introduction

The global economy, in its quest for economic efficiency during the period of extensive development (1970–2010), developed alternative materials based on polyethylene and polyurethane elastomer compounds. These compounds directly increase the marginal yield of production, but their impact on the environment and the health of the population has been neglected. In the case of water, microplastics are also found in sediments. The main characteristic of microplastics is their slow biodegradation, which leads to the formation of microplastic residues contaminating the environment and aquatic organisms. Poor aquatic wildlife health directly impacts human consumers in the food chain.

The presence of microplastics in the marine and freshwater aquatic environment has gradually increased and there is now a high rate of contamination of ecosystems and food chains, which are exposed to increasing amounts of new microplastics, hampering remediation efforts by the relevant entities.

Current research shows that the impact of microplastics on the environment, especially the aquatic environment, is devastating, leading to irreversible changes in the biodiversity of the aquatic macroenvironment and causing multiple diseases in the population.

In this context, our approach aims to demonstrate that anti-pollution policies, although heavily supported financially, are currently not very effective, particularly due to disparities in anti-pollution policies regarding types of plastic and at a regional level.

Regional disparities lead to the dissipation of efforts to combat pollution, and more effective pollution-control measures are needed.

**Figure 1:**
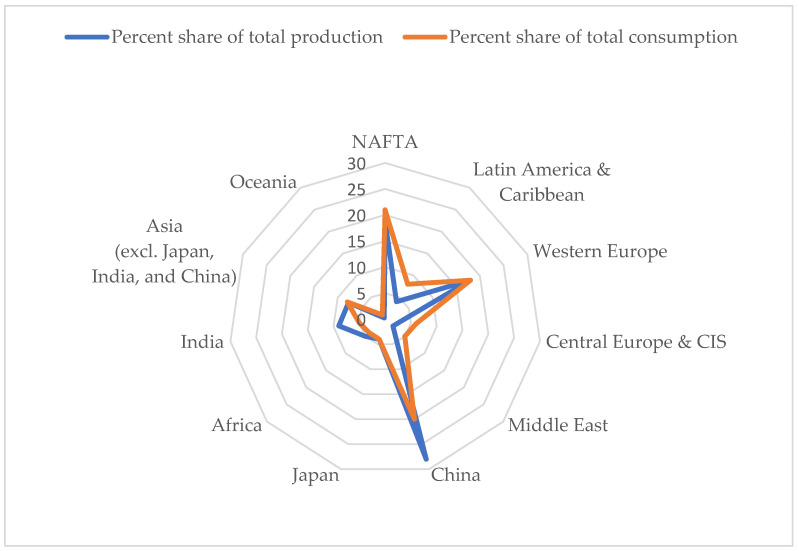
Regional disparities in microplastics consumption and production.

In the case of microplastics, both consumption and production show a large regional disparity (see Figure 1).

The authors have arrived at these disparity rates using statistics found in the United Nation’s Environment Programme, “Mapping of Global Plastics Value chain and Plastics Loses to the Environment” [1].

We started our approach with the European Commission’s implementation of the EU Plastic Strategy, which strongly emphasises the elimination of intentionally added microplastics in various products. This approach by the European Commission is supported by the European Chemicals Agency (ECHA). Unfortunately, there is no common concern in this area at the global level either, except for the relatively limited involvement of the OECD.

Returning to the EU27, some Member States have already banned by national legislation the intentional use of microplastics in consumer products. These bans cover a wide area, from food to cosmetics.

According to international statistics, 42,000 tonnes of microplastics end up in the environment every year in Europe [2]. The main sources of microplastic pollution in European waters are artificial turfs for sports fields and the wear and tear of larger pieces of plastic produced as commercial packaging waste.

In 2022, the European Commission launched a draft regulation on Registration, Evaluation, Authorisation and Restriction of Chemicals (REACH) regarding synthetic polymer microparticles [3], which came up for discussion among Member States at the end of 2022 and will be finalised in 2023.

The main directions are to set strict criteria for the release of non-degradable polymers, as the lower limit size of the particles has been eliminated.

In this case, one opposition came from the Committee for Socio-Economic Analysis (CASE) in December 2020, which supported imposing a lower limit of 1 nm for restricting microplastics.

In support of this scientific approach, we define the following research objectives:

O1. Determine the level of connection of microplastic water pollution to the regional microplastic limiting capacity;

O2. Determine the level of connection of water pollution with the level of implementation of the circular economy;

O3. Define a regional regression correlation model of microplastic water pollution control using circular economy tools;

O4. Define relevant public policy proposals to increase the effectiveness of actions to combat water pollution caused by microplastics.

The study continues with the Section 3, in which the model-building methodology is presented, followed by the Section 4 and Section 5 in which the working hypotheses and policy proposals are demonstrated. The Section 6 is dedicated to conclusions.

## 2. Literature Review

The latest research in the field of microplastic water pollution presents significant, sometimes contradictory, aspects of pollution management, sources of pollution and its impact on the environment and human health.

An extremely unpleasant finding reveals that microplastics are widely found in aquatic environments. Authors such as Shi et al. [4] look for solutions regarding the efficient removal of microplastics from water and propose nano-Fe_3_O_4_ magnetic technology, which causes optimal magnetization of microplastics by surface adsorption. The next operation consists of magnet suction. The yield of this process varies between 62% and 87% in case of water pollution with polyethylene, polypropylene, polystyrene and polyethylene terephthalate, with dimensions of about 200–900 μm.

According to Wang et al. [5], cases of advanced microplastic-contaminated water, treatment technologies, exoelectrogen biofilm and associated microbial electrochemical processes occupy an important place. The analysis quantifies the impact of microplastics on the exoelectrogenic biofilm, with potential mechanisms revealed at the gene level. The authors believe that this approach can lay the methodological foundations for the future development of efficient water treatment technologies.

Since microplastics are present in both water resources and water supply systems (in pipes), some specialists, such as Chu et al. [6], focus on quantifying the presence of these microplastics throughout the distribution system. The analysis found that nylon and polyvinyl chloride were predominant in the water samples, but that the existence of efficient drinking water treatment plants and distribution systems prevented microplastics from entering the tap water. Furthermore, the authors note the necessary correlation between the stability of pipe scales and improved water quality and safety. Monitoring the presence of microplastics in water sources is also reviewed by Nicolai et al. [7], who use a new particle counter based on a real-time fluorescence emission analysis. The case study covers polyvinyl chloride and high-density polyethylene. The presence of microplastics in a drinking water treatment plant in Barcelona (Spain) is analyzed by Dronjak et al. [8]. The analysis focuses on microplastic particles in water with sizes between 20 μm and 5 mm. The authors use Fenton’s reagent and hydrogen peroxide, as well as a zinc chloride solution. Visual identification was carried out with an optical and stereoscopic microscope, finally obtaining a microplastic removal yield of 98.3% from water, the main types of microplastics removed being polymers and synthetic cellulose, polyester, polyamide, polypropylene, polyethylene, polyurethane and polyacrylonitrile.

The conceptual approach to microplastic water pollution using reference materials is by Seghers et al. [9]. The authors support the use of a kit with microplastics immobilized in solid NaCl and a surfactant that they implemented for polyethylene terephthalate (PET) particles in water. Information on particle size distributions and shapes was obtained using laser diffraction, and the homogeneity of these particles was calculated using an ultra-microbalance.

Other authors, such as P. Wang et al. [10], propose the use of solar energy for the efficient removal of plastic particles from water. Basically, a bubble is created in a high-power density glass ball by focusing it in sunlight. It then collects the plastic particles into large clumps. The advantage of this method is that it does not use chemical or biological reagents or filters. Additionally, the costs of implementing the procedure are much lower than those of “classical” technologies.

An alarming finding is made by Karapanagioti and Kalavrouziotis [11], who state that “no microplastic removal treatment is currently used for drinking water”. The authors analysed the presence of microplastics in waters from different regions of the world, such as Russia, India, Italy, Greece and Cyprus, finding a direct connection between microplastic concentrations in water and surrounding land uses. The studies involved direct sampling of water and soil as well as sampling organisms that interacted with microplastics, such as zooplankton or zebra mussels.

An alternative method of treating water pollution containing plastic is presented by Martin et al. [12] and consists of using iron oxide nanoparticles with hydrophobic coatings to magnetize waste plastic particles. The authors claim that this method allows for the complete removal of particles 2–5 mm in size, and almost 90% of nanoplastic particles 100 nm–1000 nm in size, using a simple 2-inch NdFeB permanent magnet.

The presence of microplastics in lake waters is reviewed by Viitala et al. [13], focusing on the Lake Saimaa sub-basin (Finland). The authors quantify the connection between the presence of the local wastewater treatment plant and the plastic concentration in different compartments of the receiving lake based on the collection of bottom sediment samples. These samples were analysed using pyrolysis-gas chromatography-mass spectrometry. The results showed the presence of higher concentrations of polyethylene (PE), polypropylene (PP) and polystyrene (PS) in the water near the wastewater treatment plant effluent discharge site compared to other sites.

Microplastic pollution is more pronounced in semi-enclosed seas to which many urban conurbations have access. Authors such as Trani et al. [14] have studied the case of the Mediterranean Sea since 2012, focusing on the Salento peninsula (Apulia, Italy). The analyses cover both surface waters and microplastics ingested by certain marine organisms. For this purpose, Neuston and Manta net monitoring were used and the level of microplastic contamination of different fish and mussel species was targeted. The results of the analysis show that microplastic water pollution is higher in the Adriatic Sea than in the Ionian Sea and that the concentration of microplastics at the sea surface and in the gastrointestinal tract of targeted species is higher. Another semi-enclosed sea is the Black Sea, whose plastic pollution is analysed by Strokal et al. [15] based on five scenarios modelled using a model assessing riverine inputs of pollutants to the sea (MARINA-Global) for 107 sub-basins. The authors state that European rivers flowing into the Black Sea discharge more than half of all microplastics and, as a result, make proposals for environmental policies capable of reducing pollution in the Black Sea to zero. Microplastic pollution of marine systems is the subject of an investigation by Yuan et al. [16] that reviews the current state of research in this area. The authors consider seafood consumption, lung inhalation and skin infiltration to be the main causes of human exposure to microplastics from the marine environment. The authors highlight the risks that microplastics in water pose on human health, referring to certain cancers and chronic and acute toxicity. The risks microplastics pose to human health are also addressed by Sarma et al. [17]. The authors conclude that urban wastewater flushing is the main source of microplastic water pollution. The impact of microplastics on human health through commercial fish, crustacean and bivalve species, is addressed by Sánchez-Guerrero-Hernández et al. [18], based on a case study of the main commercial fish species in Spain: the European anchovy and the European sardine. In order to determine the presence of microplastics in these fish, the authors used an alkaline organic oxidant (KOH-H_2_O_2_), which identified nylon as the main polymer found in both fish species. The impact of microplastics on health is the subject of a study by Kadac-Czapska et al. [19], who consider that the most common route of exposure is the gastrointestinal tract. In this context, microplastics (PET, PE, PP, PS, PVC, PA and PC) enter the human body through the consumption of fish, shellfish and water.

Technologies to remove microplastics from water are investigated by Gao et al. [20], who consider both technical processes and related costs. Furthermore, the authors refer to the practical efficiency of plastic removal technologies from water and their impact on the environment.

Microplastic pollutants < 5 mm in diameter from different countries and regions are reviewed by Yang et al. [21] in terms of abundance, morphology and polymer types in water and lake sediments. The authors conclude that the level of microplastic pollution depends on the level of local development and the economic structure of the areas analysed. The authors sound the alarm for an optimal microplastic pollution control system in lake systems.

The connection between microplastics (polyethylene and polyvinyl chloride), UV and bacteria (Gram-negative and Gram-positive) in water is presented by Manoli et al. [22]. The authors aim to quantify the effect of microplastics on UV disinfection performance in order to increase the efficiency of physical and chemical disinfection processes in different waters.

The increase in plastic production, the development of international trade in these products and plastic waste and the intensification of the use of plastics in the economy on the African continent are addressed by Deme et al. [23]. The authors study legislation supporting sustainable economic development in African countries and conclude that national policymakers’ approaches are ineffective in this area. As a result, these authors support environmental policy decisions based on price, legislation and the implementation of the best practices in microplastics waste management.

An interesting analysis developed by Usman et al. [24] discusses plastic production, plastic waste management defects and human health. The analysis shows that the presence of microplastics in food and drinking water has long-term health effects on the population. The authors mention that “there is no regulation of plastic contamination of food and drinking water” and propose increased collaboration in this area at international and national levels. Microplastic water pollution can lead to rare forms of cancer, as shown in a study by Mocanu et al. [25]. This approach is also taken up in the research carried out by Nastase et al. [26].

Lofty et al. [27] critique the circular economy from the perspective of the use of sewage sludge generated by wastewater treatment plants in agriculture. The authors believe that there is a possibility that plastic successfully removed from sewage treatment plants and deposited in the soil may return to natural waters through runoff or seepage into groundwater. Based on official statistics provided by the European Commission and Eurostat, the authors state that the practice of spreading sludge on agricultural land can lead to the creation of an impressive global reservoir of plastic pollution. A contrary approach sees the circular economy as the key to a more sustainable use of plastic. The authors of this approach, Syberg et al. [28], consider that “explicit considerations of microplastics contamination are rarely taken into account in studies of the transition to a plastic circular economy”. Furthermore, they state that there are situations and areas where recycling can lead to increased microplastic contamination. The circular economy from the perspective of microplastic water contamination is addressed by Syberg et al. [28], with the authors providing recommendations on how reducing microplastic contamination and transitioning to the circular economy can be interlinked in future research. Moreover, in the view of Cook et al. [29], the development of the circular economy must not have negative effects on human health and the environment. The authors use the scenario method to quantify the environmental impact of post-consumer plastic packaging resource recovery processes and recommend to developing countries the mechanical reprocessing of these plastics at the expense of chemical recycling procedures. In the framework of the circular economy, bioplastics represent a great challenge according to Rosenboom et al. [30] in the process of transforming them into high-quality materials. The authors stress the need for new regulations and financial incentives to support the sustainable recycling of these categories of bioplastics.

Perpetuation of plastic pollution along the food chain in the aquatic environment of the Vipacco River, northeastern Italy is studied by Bertoli et al. [31], who state that the main source of microplastic pollution in the aquatic environment is urban wastewater discharge. The effects of this pollution are quantified at the level of entire macrobenthic invertebrate communities and have results that are difficult to generalize. As a result, the authors stress the need for further studies. In this context, other authors, such as Mehinto et al. [32], propose a risk-management system for aquatic ecosystems. The authors establish four thresholds for plastic contamination of water based on studies in the literature, on the basis of which they define two mechanisms of effects: dietary dilution with thresholds ranging from ~0.5 to 35 particles/L and tissue translocation with thresholds ranging from ~60 to 4100 particles/L. Another model for risk assessment of marine water pollution with plastic is presented by Yuan et al. [33], who call for a screening strategy. This strategy allows the prioritisation of polymers of primary interest in marine waters: PUR, PVC, PAN, ABS, PMMA, SAN, TPU, UP, PET, PS and HDPE. The authors make recommendations to policy makers on how to better manage microplastics in marine waters. Microplastics are, according to Hossain et al. [34], one of the fastest-growing wastes in the world. The authors conduct an impressive meta-analysis of Australia’s plastic waste management system in the context of the transition to the circular economy. The analysis shows that the most widespread forms of plastic in the environment are high-density polyethylene, polyethylene terephthalate and low-density polyethylene. In the case of microplastics, households generate the largest amount of PET and HDPE. The management of microplastic waste, including that found in water, is strongly influenced by the involvement of local and regional communities.

According to research by Campanale et al. [35], 50% of microplastic particles between 0.02 and 0.1 mm in size are transported by water runoff. The authors focus their research on temporary ponds, stormwater retention ponds and small streams, drawing attention to the extremely small number of studies (eight) conducted so far on the ecosystems and related to these water resources.

Other authors, such as Vuori and Ollikainen [36], point out that there are no standards regulating the amount of microplastics in wastewater. Their approach focuses on the cost-effectiveness of three types of wastewater treatment (activated sludge, rapid sand filtration and membrane bioreactor) and two sludge management technologies (anaerobic digestion and incineration), aiming to quantify the impact of microplastic pollution on the aquatic environment and aquatic ecosystems. The analysis concludes that the removal of microplastics from wastewater is technically feasible and economically profitable.

An interesting cause of increased water pollution caused microplastics is the impact of flooding on waste management facilities. According to Ponti et al. [37], these floods can release micropollutants into freshwater systems, impacting the marine environment, agricultural ecosystems and human health. Based on the existing situation in the UK, the authors propose a correlated analysis of the official waste statistics with rainfall and river flood extent maps. Furthermore, they believe that site-specific mitigation measures and containment systems capable of reducing the amount of flood-induced microplastics from waste management facilities are needed.

Risk management for aquatic ecosystems is considered by Mehinto et al. [32] to be closely related to pollution control measures that mitigate environmental emissions. The authors use four pollution risk thresholds, official statistical data, microplastic toxicity studies and a metanalysis in the field. Following this analysis, the authors make recommendations on the quantification of water pollution, including microplastics, and a more efficient identification of risk thresholds. Risk management of microplastic pollution of water sources is also addressed by Thornton Hampton et al. [38], who point out that there is no internationally unified approach to how microplastic concentrations should be reported. For this reason, the authors recommend that microplastic concentrations should be calculated at least by both mass and number.

An analysis of the degree of pollution of water sources caused by microplastics is carried out by Chakraborty et al. [39] and covers the period of 2015–2021. The authors use Raman spectroscopy and conclude that the most widespread microplastics in water sources are polystyrene (PS), polyethylene terephthalate (PET), polyethylene (PE) and polypropylene (PP). In addition to this, the authors define the main sources of microplastic water pollution as urban waste, fishing activities and industrial waste.

An interesting study carried out by Angelakis et al. [40] highlights, on the one hand, the historical evolution of water quality and, on the other hand, the current challenges for water quality management and protection. The authors believe that the analysis of the methods and solutions offered by the evolution of mankind in relation to the management of water sources is beneficial to look at for present and future solutions in this field.

The analysis of microplastic pollution in Italian marine waters is carried out by Sbrana et al. [41], in the context of European marine water protection legislation and its impact on marine ecosystems. The analysis reveals that the concentration of microplastics in the water decreases with distance away from the coast, except in areas where sea currents are very strong. Moreover, the concentration of plastic in surface waters is four times higher than in deep waters.

In the case of the Lis river basin (Portugal) and coastal area, microplastic pollution is analysed by Sá et al. [42], the authors using a sample of 105 companies in the area and comparing samples collected from surface water and sediment. The most common particles in the water analysed were polyethylene (37%), polyacrylate (18%) and polystyrene (18%), and in sediments, polyethylene terephthalate (29%) and polyacrylate (23%). The analysis concludes that factors contributing to the increase in microplastic water pollution are population growth, plastic production and environmental conditions conducive to the transmission of microplastic particles into water sources. A similar approach, carried out by Kittner et al. [43], considers microplastic pollution in the Danube River and aims to define a systematic pollution-monitoring strategy. Chemical analysis is performed using the thermal extraction desorption technique, gas chromatography/mass chromatography. Following the analysis of the collected samples, polyethylene, polystyrene and polypropylene were, alarmingly, found in abundance in the water.

The lack of standard protocols and technologies for removing microplastics from water through wastewater treatment plants is addressed by Sadia et al. [44], who review the efficiency of wastewater treatment plants and the possibility of converting microplastics into renewable energy sources. To this end, the authors developed a sustainable methodology for wastewater treatment.

Other authors such as Melchor-Martínez et al. [45] stress the need for sustainability of microplastic production under conditions of increasing economic efficiency. The authors conducted a meta-analysis of production methods, highlighting the environmental impact and mitigation of conventional and emerging plastics, as well as regulations in the field.

In terms of plastic recycling, according to Nikiema and Asiedu [46], only 9% of the 9 billion tonnes of plastic ever produced has so far been recycled. The authors reviewed microplastic removal technologies and their efficiency, starting with pollution sources and until microplastics reach the sea, covering stormwater, municipal wastewater treatment and drinking water. The final result provides a guide on implementable measures for the treatment and elimination of water pollution caused by microplastics.

A new technology for removing plastics from water sources is the use of superhydrophobic surfaces, which have a water contact angle of >150°. According to Rius-Ayra et al. [47], the increase in research related to this technology shows its importance. The authors believe that superhydrophobic materials allow the removal of five types of emerging pollutants, including microplastics.

The literature review is an argument in favour of the present scientific approach and highlights the need for a new approach in the field.

## 3. Materials and Methods

Since the intensification of global trade, interventions to limit environmental pollution caused by microplastics, especially in the aquatic environment, have become a priority for international environmental organisations. Several campaigns have been carried out to monitor pollution and inform stakeholders about its effects.

These issues have taken on a global dimension through OECD efforts to collect information on microplastic pollution. In our scientific research, we used OECD databases [48] for the period of 1990–2019 for the indicators presented in Table 1.

Based on the collected data and literature review, we formulated the following research hypotheses:

**H1.**  *At the global level, the policy to combat water pollution caused by microplastics is directly and proportionally oriented towards the reduction of regional pollution, with the awareness that this approach will have an effect of at least 98% in the total reduction in water pollution caused by microplastics. The hypothesis is a continuation of the results of the research by the authors Blanco et al., Chu et al., Dronjak et al., Karapanagioti and Kalavrouziotis, Nicolai et al., Seghers et al., Shi et al., P. Wang et al. and S. Wang et al. [4,5,6,7,8,9,10,11,53].*

**H2.**  *Globally, plastic recycling mechanisms have been set up on the assumption that this will have a direct impact on reducing microplastic water pollution. The definition of this hypothesis was made in accordance with the results of the research undertaken by the authors Angelakis et al., Chakraborty et al., Kittner et al., Mehinto et al., Sá et al. and Thornton Hampton et al. [32,38,39,40,42,43].*

**H3.**  *From the point of view of the coherence of water pollution reduction policies, there is an increasing trend in the dynamics towards the reduction in correlation errors of the indicators as the overall experience of the implementation of these policies increases. The construction of this hypothesis was based on research conducted by the authors Melchor-Martínez et al., Nikiema and Asiedu, Rius-Ayra et al. and Sadia et al. [44,45,46,47].*

**H4.**  *At the EU level, against the background of intensified efforts to promote the circular economy, the disparities in terms of combating water pollution caused by microplastics are widening, especially for countries where the implementation of the circular economy is at an early stage. This hypothesis is also supported by research carried out by the authors Cook et al., Lofty et al., Rosenboom et al. and Syberg et al. [27,28,29,30].*

Using data reported by the OECD (Table 1), we performed a multiple regression correlation diagram for 15 regions in the world. It has as its pivot the dependent variable assimilated to the circular economy, i.e., the amount of plastic waste collected for recycling, which we treated in relation to the monitoring indicators of plastic waste produced at the regional level, end-of-life plastic waste and the impact of plastic pollution on the aquatic environment.

The system of regional equations for the variables in Table 1 is presented as follows:

(1) $$\left\{ \begin{array}{c} PWCR_{US}=1.693*PLAE_{US}+0.043*PUR_{US}+0.068*PWRELF_{US}-0.742\\ PWCR_{CAN}=1.448*PLAE_{CAN}-0.086*PUR_{CAN}+0.143*PWRELF_{CAN}-0.111\\ PWCR_{OECDAmerica}=1.003*PLAE_{OECDAmerica}-0.173*PUR_{OECDAmerica}+0.236*PWRELF_{OECDAmerica}+0.14\\ PWCR_{OECDEU}=3.874*PLAE_{OECDEU}-0.158*PUR_{OECDEU}+0.2*PWRELF_{OECDEU}-1.7\\ PWCR_{OECDNonEU}=-0.021*PLAE_{OECDNonEU}-0.272*PUR_{OECDNonEU}+0.509*PWRELF_{OECDNonEU}-0.299\\ PWCR_{OECDASIA}=0.094*PLAE_{OECDASIA}-0.275*PUR_{OECDASIA}+0.592*PWRELF_{OECDASIA}+0.115\\ PWCR_{OECDOceania}=21.215*PLAE_{OECDOceania}-0.009*PUR_{OECDOceania}-0.204*PWRELF_{OECDOceania}+0.003\\ PWCR_{OECDLatAmerica}=1.644*PLAE_{OECDLatAmerica}+0.084*PUR_{OECDLatAmerica}-0.175*PWRELF_{OECDLatAmerica}-0.083\\ PWCR_{OthEU}=11.154*PLAE_{OthEU}-0.014*PUR_{OthEU}-0.141*PWRELF_{OthEU}+0.045\\ PWCR_{OthEurasia}=4.762*PLAE_{OthEurasia}+0*PUR_{OthEurasia}-0.03*PWRELF_{OthEurasia}-0.025\\ PWCR_{MENorthAfrica}=1.096*PLAE_{MENorthAfrica}-0.029*PUR_{MENorthAfrica}-0.005*PWRELF_{MENorthAfrica}+0.065\\ PWCR_{OthAfrica}=0.611*PLAE_{OthAfrica}+0.073*PUR_{OthAfrica}-0.105*PWRELF_{OthAfrica}-0.06\\ PWCR_{NonOECDASIA}=1.06*PLAE_{NonOECDASIA}-0.037*PUR_{NonOECDASIA}-0.048*PWRELF_{NonOECDASIA}+0.156\\ PWCR_{China}=5.051*PLAE_{China}+0.05*PUR_{China}-0.158*PWRELF_{China}-0.525\\ PWCR_{India}=5.577*PLAE_{India}+0.035*PUR_{India}-0.226*PWRELF_{India}-0.029 \end{array}\right.$$

**Figure 2:**
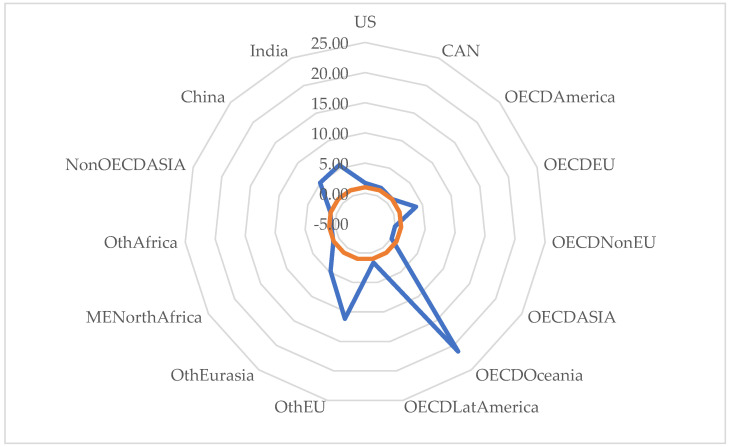
Correlation diagram of the effects of the implementation of the circular economy on the reduction of water pollution caused by microplastics (PWCR vs. PLAE).

From the analysis of the system of equations constructed on the basis of the β coefficients of the 15 regional models, it is evident that the variable that best correlated with the outcome indicator of circular economy efficiency (dependent variable) is the indicator of monitoring the impact of plastic pollution on the aquatic environment. Thus, the impact of the circular economy results in long-term effects on the reduction of microplastic pollution, especially in China, India, Oceania and Europe (see Figure 2).

The diagram shows that, at the level of the performing sample, the average correlation is 500%, i.e., the impact of reducing environmental plastic pollution generates a 5-fold reduction in the impact of microplastics on the aquatic environment in the circular economy, the maximum magnitude belonging to the Oceania region, where the impact is 21 times greater. At the European level, in OECD member countries, the impact is up to 3.8 times and up to 11 times in non-OECD member countries.

Non-EU countries show the lowest correlation between the two indicators, with an inversely proportional variation of 0.21%. This means that, in these countries, the impact of the circular economy is low and the strategies to reduce microplastic pollution are not in accordance with the global and European action guidelines.

**Figure 3:**
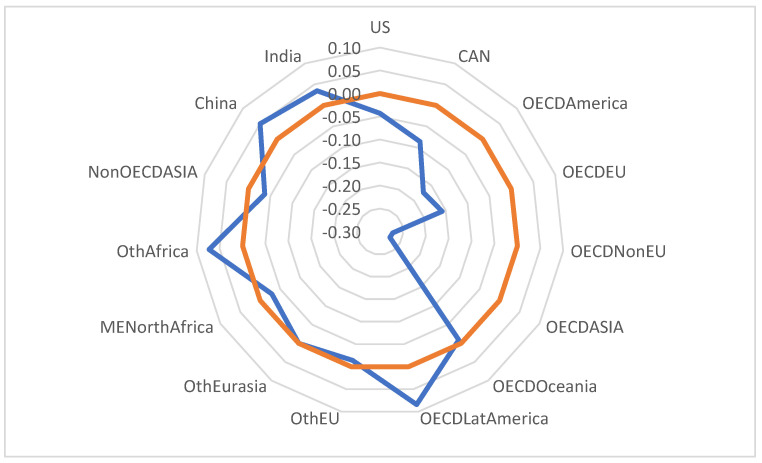
Correlation diagram of the effects of circular economy implementation on plastic use by region (PWCR vs. PUR).

After using a multiple regression correlation at the regional level (see Figure 3), it was observed that there is an inversely proportional relationship between plastic consumption and use in the regional circular economy of the USA, Canada, OECD America, OECD EU, OECD Non-EU, OECD Asia, OECD Oceania, Other EU, ME North Africa and non-OECD Asia. The average value of the inverse correlation is 10%. For the other regions analysed in the sample, the correlation is directly proportional, but reduced by a maximum of 8%, which shows that, in relation to the development objectives of clean industry, plastic consumption does not show significant changes. We consider this a major vulnerability of pollution policies, which, in this light, should focus additional efforts to improve public policies.

**Figure 4:**
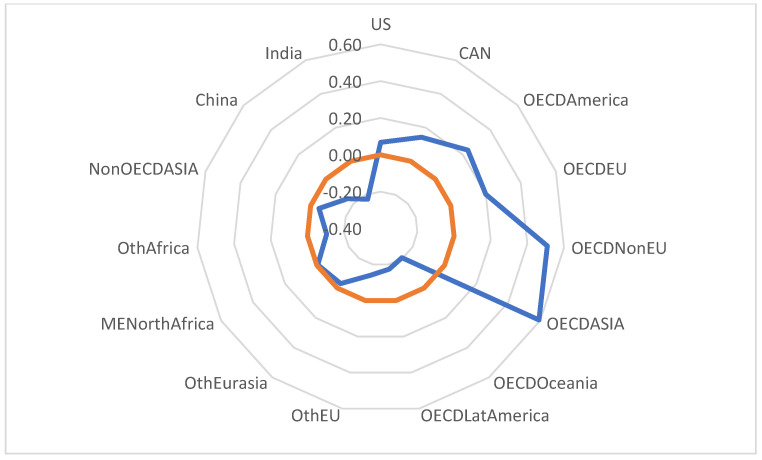
Correlation diagram of the effects of the implementation of the circular economy on end-of-life plastic waste (PWCR vs. PWRELF).

Figure 4 shows a significant disparity in the correlation between the implementation of the circular economy and the reduction of the mass of end-of-life plastic waste, where developed regions of the world (USA, Canada, OECD America, OECD EU, OECD Non-EU, OECD Asia) have higher correlation rates. These regions have access to superior technologies to combat microplastic pollution according to its chemical properties with significant biological impact. The average correlation reaches 30%, with a maximum of 60% in the region of Asian OECD member states.

In the other regions analysed, the correlation is inversely proportional, which means that the policy to combat microplastic pollution does not meet the proposed goal, one explanation being the orientation of these regions towards commercial expansion and extensive economic growth.

## 4. Results

Through the application of modelling techniques, the regional model was summarized, which generated statistically significant coefficients for all 15 regions studied, with the level of statistical significance exceeding 98% for these regions, with a standard maximum error estimate of 8% at the level of the sample studied, as shown in Table 2.

This validates hypothesis H1. At the global level, the policy to combat microplastic water pollution is directly and proportionally oriented towards the reduction of regional pollution, with the awareness that this approach will have an effect of at least 98% in the total reduction of microplastic water pollution.

Table 2 shows the distribution of the statistical F function values between the minimum value of 236.93 points and the maximum value of 5058.97 points, thus demonstrating a significant regional disparity of policies to combat microplastic water pollution. The maximum values are attributed to the regions: USA, OECD Latin America, Other Africa and non-OECD Asia. With respect to these regions, the analysis of the interval between 1990–2019 shows that the value of the circular economy’s impact on microplastic water pollution reaches the maximum value at the correlation function. 

At the opposite pole, the lowest values were recorded for the regions: OECD America, OECD Non-EU and OECD Asia. It should be mentioned that the level of Sig. coefficients assimilated to the F function is lower than the selected error representativeness threshold α = 0.05, which allows for all 15 regions analyzed to reject the null hypothesis and maintain the alternative hypothesis. This allows the validation of the regional model to combat microplastic pollution. The ANOVA test is presented in Table 3.

The ANOVA statistical test for the regional models shows the statistical weight of the regression squares is allocated to the correlational function (98.5%), while the residual variable has an allocation of only 99.5%. This demonstrates that the model is valid and representative of the phenomenon studied, validating hypothesis H2. At the global level, plastic recycling mechanisms were created assuming that this approach will have a direct impact on reducing microplastic water pollution.

**Figure 5:**
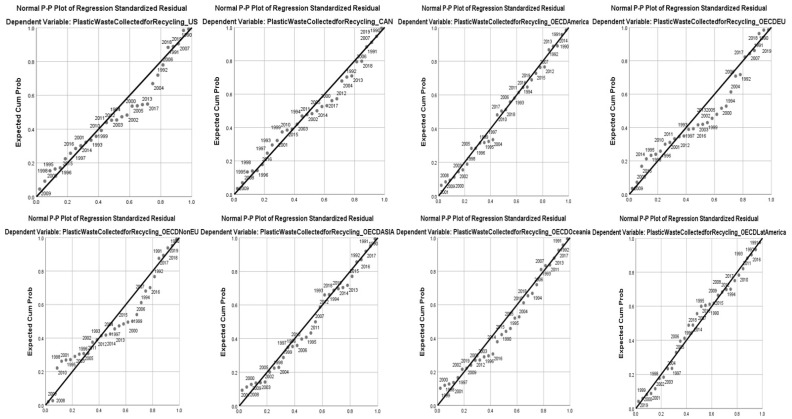

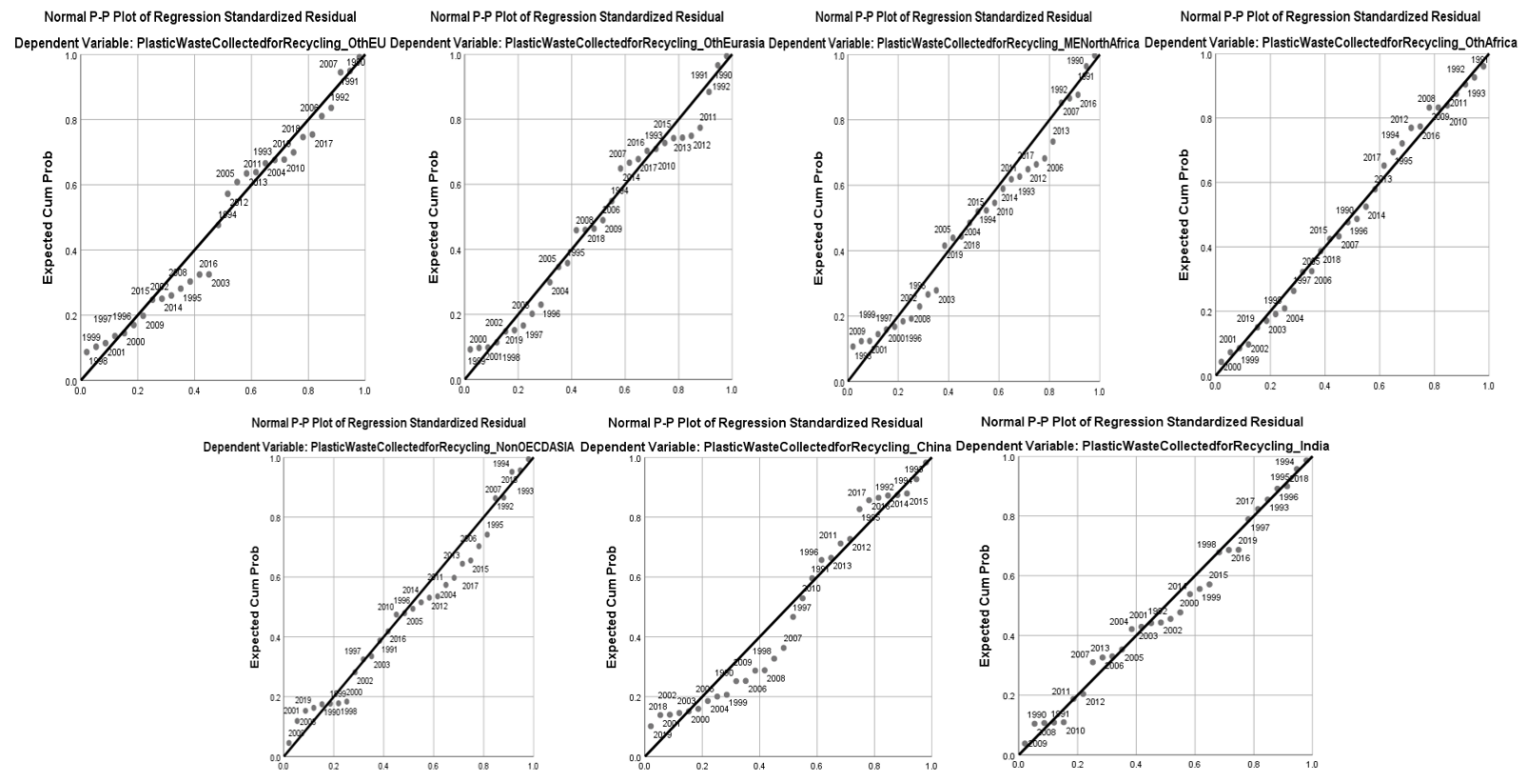
P-P Plot diagrams of regional distribution.

To prove hypothesis H3, we projected in Figure 5 the dynamic distributions of the evolution of the dependent variable in relation to its predicted values according to the regional PP-Plot distribution graph. From Figure 5, it follows that the error distribution of the dependent variable at the regional level has different error peaks ($\bar{y}$).

Figure 5 shows that, as experience increases and the current period approaches, the experience gained by policy makers in the field of pollution control helps straighten the trend curves, which proves hypothesis H3. From the point of view of coherence of water pollution abatement policies, there is an increasing trend to reduce the correlation errors of the indicators as the overall experience of implementing these policies increases.

In order to prove hypothesis H4, the authors analysed the results obtained by the proposed model (Equation (1)), finding that, at the level of the dependent variable assimilated to the circular economy, the correlation with the impact of water pollution caused by microplastics was strong, i.e., reducing the amount of plastic waste has the effect of reducing water pollution caused by microplastics by 3.8 times. It can be seen from equation 1 that the impact of the circular economy on the regional size of plastic consumption in the EU is inversely proportional, which can be presumed to have a causality with the high level of regional disparity in plastic consumption. In order to determine the level of regional disparity of plastic use in the EU, we accessed the Our World in Data database [54] for the year 2019 (the latest year for which official statistical data are available) and selected Member States for which we performed an algorithm to plot regional averages against the overall average for the following indicators: share of EU average mismanaged plastic waste (%); share of EU average mismanaged plastic waste to ocean (%) and share of EU average mismanaged plastic waste per capita (%) (see Table 4).

The overall pollution disparity index calculated based on the standard deviation of the three regional data sets in Table 4 is 65%. Thus, there is a significant difference between policies to reduce plastic water pollution and policies to reduce plastic consumption per capita in the EU. At a regional level, the share of the EU average mismanaged plastic waste indicator has a disparity of 136%, close to the value of the disparity of the share of the EU average mismanaged plastic waste per capita indicator, which means that, in terms of communicating the effects on the environment, there is a successful communication effort in the EU, with 90% of European citizens aware of the consequences of pollution on the deterioration of environmental quality. This results from a comparison of regional disparities between the two indicators.

On the other hand, the Share of EU average mismanaged plastic waste to ocean indicator shows an increase in regional disparities, up to 169.2%, mainly due to the regional territorial configuration. The most advanced countries in implementing environmental policies in this area are Malta, Finland, Ireland and Slovakia. At the opposite end are Croatia, Greece, the Netherlands, Estonia and Portugal.

In the overall ranking for the three indicators, the highest levels of disparity were assessed for Croatia, Germany and Romania, with these countries showing the most fluctuating variations from the calculated EU average. These aspects demonstrate hypothesis H4. In the EU, as efforts to promote the circular economy intensify, the disparities in combating water pollution caused by microplastics are widening, especially for countries where the implementation of the circular economy is at an early stage.

## 5. Discussion

The research results allow the following aspects to be presented:

- In the US, the synergy between anti-pollution policies and the acceleration of the circular economy is marked by the period 2004–2013, which covers the global recession of 2007–2008, with a significant impact on the US economy;
- The period 2004–2012 was the impact period for Canada’s economy, which takes on the effects of the US crisis;
- In OECD countries in the Americas, there was a reduction in errors relating to the projection of forecast values in relation to the trend line, the peaks being characterized by the period 1997–2004. This was against the backdrop of the terrorist attacks of 11 September 2001, followed by the stock market crisis and the effects of the war in Iraq in 2011–2003. These events redirected public policies towards security, minimising efforts for other policies such as pollution control;
- With 22 EU OECD member states, the distribution of errors is significant when compared to correct forecast rates and covers extended periods, namely 1992–1995, 2000–2004 and 2010–2014. In the first mentioned timeframe, major changes in the European economic policy occurred as a result of the fall of the communist regime in SE Europe. The second timeframe was assimilated to the EU enlargement efforts, which culminated in 2004 with the accession of 10 former communist states to the European bloc. The 2010–2014 timeframe represents the period of global recession triggered in 2007 in the US and rapidly propagated in the EU. This was a period that saw the bankruptcy of some renowned European institutions and the reshaping of European policies on financial risks and stock market trading risks. The phenomenon was boosted by the last two EU enlargements in 2007 and 2013;
- The distribution of errors was also transferred to the OECD Non-EU countries in the region, which, in the periods 1997–2000, 2005–2007 and 2010–2016, faced problems accessing the European single market and structural changes in trade due to EU regional reconfiguration;
- For Asian OECD member countries, the distribution of trend curves in relation to predicted values for the circular economy-like variable is superior, with more efficient correlations. The main disruptions the took place in 1995–2000 and 2011–2014. In the first period, the economies of these countries were heavily affected by the major financial crisis that began in Thailand involving the link between the Thai baht and the U.S. dollar. Indonesia, South Korea and Malaysia were involved in this crisis and their capital inflows were affected by more than $100 billion in the first year of the crisis. The effects of the Asian financial crisis spilled over into the economies of Russia and Brazil. The second period was a consequence of the global economic crisis that started in 2008;
- In Oceania’s case, the values for the distribution of errors relative to the trend curve are flattened, the significant periods being 1990–1995 and 2012–2016;
- In the case of OECD Latin American countries, no significant error distributions were found, with the right-hand side of the forecast containing the likely values of the regression function;
- In the case of other non-OECD European countries, error distributions were found for the periods 1996–1999 (the fall of the communist regime and its consequences) and 2003–2014. These periods coincided with the reconfiguration of EU borders and the major global crisis of 2009;
- Non-OECD Eurasia shows the most significant errors distributed over the period 2011–2013, corresponding to the global crisis of the early 2010s;
- In the non-OECD African region, there are no significant distributions of errors to the right of the trend. The model is representative of this region;
- In non-OECD Asian countries, the period that witnessed function errors is clustered between 2012 and 2017 and was strongly influenced by the effects of the economic crisis in China in 201;
- At the level of the Chinese economy, there is a significant distribution of errors to the right of the trend for several periods, namely: 1990–1995, 1997–1999 and 2004–2009;

The Indian economy was characterized by significant error distribution in the periods of 1999–2000 and 2015–2019,

Based on the results of the analysis, we formulated the following proposals to improve public policies in the field of combating water pollution caused by microplastics (see Table 5).

The importance of this study lies in combining in an integrated way OECD data on microplastic water pollution and identifying relevant policies to combat this type of pollution.

## 6. Conclusions

The authors have achieved the objectives proposed in the research as defined in the introduction. The literature review allowed the development of research hypotheses, which were subsequently demonstrated in Section 3 and Section 4 of the paper.

An interesting econometric model was built to study the connection between the factors contributing to the increase of microplastic water pollution. Following the implementation of this model at the OECD database level, proposals for improving public policies in the field of combating water pollution caused by microplastics were carried out.

The novelty of the econometric model and public policy proposals for decreasing water pollution caused by microplastics are the strengths of this scientific approach. The novelty of the approach lies in linking chemical elements with economic and social impacts at a regional level. The model is entirely unprecedented and is in no way an adaptation or update of an existing model. The public policy proposals in this area were also innovative.

This scientific work encountered difficulties, including the lack of official statistical information on water pollution caused by microplastics and the lack of a unified approach and common regulations at an international level.

The main limitation of this paper is the relatively small number of indicators used in the analysis. The authors propose to expand the number of indicators analysed and the statistical basis of observations through international collaboration, at least at the academic level, in a future scientific approach on the same topic.

## Figures and Tables

**Table 1:** Description of the indicators used in the analysis.

Region	Symbol	Name	Unit of Measure	Databases (accessed on 3 January 2023)
United States (US) Canada (CAN) OECD America OECD EU OECD non-EU OECD Asia OECD Oceania OECD Latin America (OECDLatAmerica) Other non-OECD EU (OthEU) Other Eurasia (OthEurasia) Middle East North Africa (MENorthAfrica) Other Africa (OthAfrica) Non-OECD Asia (NonOEDASIA) China India	PWCR	plastic waste collected for recycling	tonnes (t) of plastics	https://stats.oecd.org/viewhtml.aspx?datasetcode=PLASTIC_WASTE_6&lang=en [49]
	PLAE	plastic leakage to aquatic environments		https://stats.oecd.org/viewhtml.aspx?datasetcode=PLASTIC_LEAKAGE_5&lang=en [50]
	PUR	plastics use by region		https://stats.oecd.org/viewhtml.aspx?datasetcode=PLASTIC_USE_9&lang=en [51]
	PWRELF	plastic waste by region and by end-of-life fate		https://stats.oecd.org/viewhtml.aspx?datasetcode=PLASTIC_WASTE_5&lang=en [52]

**Table 2:** Model Summary.

Model	R	R Square	Adjusted R Square	Std. Error of the Estimate	Change Statistics					Durbin-Watson
					R Square Change	F Change	df1	df2	Sig. F Change	
1.	0.999 ^a,b^	0.997	0.997	0.099143	0.997	2992.770	3	26	0.000	0.715
a. Predictors: (Constant), PWRELF_US_, PUR_US_, PLAE_US_										
b. Dependent Variable: PWCR_US_										
2.	0.998 ^a,b^	0.997	0.997	0.013844	0.997	2857.987	3	26	0.000	0.831
a. Predictors: (Constant), PWRELF_CAN_, PUR_CAN_, PLAE_CAN_										
b. Dependent Variable: PWCR_CAN_										
3.	0.994 ^a,b^	0.988	0.987	0.054194	0.988	727.162	3	26	0.000	0.456
a. Predictors: (Constant), PWRELF_OECDAmerica_, PUR_OECDAmerica_, PLAE_OECDAmerica_										
b. Dependent Variable: PWCR_OECDAmerica_										
4.	0.998 ^a,b^	0.995	0.995	0.285726	0.995	1822.884	3	26	0.000	0.608
a. Predictors: (Constant), PWRELF_OECDEU_, PUR_OECDEU_, PLAE_OECDEU_										
b. Dependent Variable: PWCR_OECDEU_										
5.	0.991 ^a,b^	0.982	0.980	0.099378	0.982	465.494	3	26	0.000	0.543
a. Predictors: (Constant), PWRELF_OECDNonEU_, PUR_OECDNonEU_, PLAE_OECDNonEU_										
b. Dependent Variable: PWCR_OECDNonEU_										
6.	0.982 ^a,b^	0.965	0.961	0.156510	0.965	236.939	3	26	0.000	0.254
a. Predictors: (Constant), PWRELF_OECDASIA_, PUR_OECDASIA_, PLAE_OECDASIA_										
b. Dependent Variable: PWCR_OECDASIA_										
7.	0.998 ^a,b^	0.997	0.997	0.003728	0.997	2865.476	3	26	0.000	1.108
a. Predictors: (Constant), PWRELF_OECDOceania_, PUR_OECDOceania_, PLAE_OECDOceania_										
b. Dependent Variable: PWCR_OECDOceania_										
8.	0.999 ^a,b^	0.997	0.997	0.047812	0.997	3143.947	3	26	0.000	0.396
a. Predictors: (Constant), PWRELF_OECDLatAmerica_, PLAE_OECDLatAmerica_, PUR_OECDLatAmerica_										
b. Dependent Variable: PWCR_OECDLatAmerica_										
9.	0.998 ^a,b^	0.996	0.996	0.004837	0.996	2360.164	3	26	0.000	0.491
a. Predictors: (Constant), PWRELF_OthEU_, PUR_OthEU_, PLAE_OthEU_										
b. Dependent Variable: PWCR_OthEU_										
10.	0.998 ^a,b^	0.996	0.995	0.027975	0.996	1973.765	3	26	0.000	0.177
a. Predictors: (Constant), PWRELF_OthEurasia_, PUR_OthEurasia_, PLAE_OthEurasia_										
b. Dependent Variable: PWCR_OthEurasia_										
11.	0.998 ^a,b^	0.996	0.995	0.027183	0.996	2052.844	3	26	0.000	0.454
a. Predictors: (Constant), PWRELF_MENorthAfrica_, PUR_MENorthAfrica_, PLAE_MENorthAfrica_										
b. Dependent Variable: PWCR_MENorthAfrica_										
12.	0.999 ^a,b^	0.998	0.998	0.019682	0.998	4091.570	3	26	0.000	0.362
a. Predictors: (Constant), PWRELF_OthAfrica_, PLAE_OthAfrica_, PUR_OthAfrica_										
b. Dependent Variable: PWCR_OthAfrica_										
13.	0.999 ^a,b^	0.998	0.998	0.047965	0.998	5058.977	3	26	0.000	1.023
a. Predictors: (Constant), PWRELF_NonOECDASIA_, PUR_NonOECDASIA_, PLAE_NonOECDASIA_										
b. Dependent Variable: PWCR_NonOECDASIA_										
14.	0.998 ^a,b^	0.996	0.996	0.275743	0.996	2227.735	3	26	0.000	0.389
a. Predictors: (Constant), PWRELF_China_, PLAE_China_, PUR_China_										
b. Dependent Variable: PWCR_China_										
15.	0.998 ^a,b^	0.996	0.995	0.074138	0.996	2115.586	3	26	0.000	0.294
a. Predictors: (Constant), PWRELF_India_, PLAE_India_, PUR_India_										
b. Dependent Variable: PWCR_India_										

**Table 3:** ANOVA test.

Model		Sum of Squares	df	Mean Square	F	Sig.
1.	Regression ^a,b^	88.251	3	29.417	2992.770	0.000 ^b^
	Residual	0.256	26	0.010		
	Total	88.507	29			
a. Dependent Variable: PWCR_US_						
b. Predictors: (Constant), PWRELF_US_, PUR_US_, PLAE_US_						
2.	Regression ^a,b^	1.643	3	0.548	2857.987	0.000 ^b^
	Residual	0.005	26	0.000		
	Total	1.648	29			
a. Dependent Variable: PWCR_CAN_						
b. Predictors: (Constant), PWRELF_CAN_, PUR_CAN_, PLAE_CAN_						
3.	Regression ^a,b^	6.407	3.000	2.136	727.162	0.000 ^b^
	Residual	0.076	26.000	0.003		
	Total	6.483	29.000			
a. Dependent Variable: PWCR_OECDAmerica_						
b. Predictors: (Constant), PWRELF_OECDAmerica_, PUR_OECDAmerica_, PLAE_OECDAmerica_						
4.	Regression ^a,b^	446.456	3.000	148.819	1822.884	0.000 ^b^
	Residual	2.123	26.000	0.082		
	Total	448.578	29.000			
a. Dependent Variable: PWCR_OECDEU_						
b. Predictors: (Constant), PWRELF_OECDEU_, PUR_OECDEU_, PLAE_OECDEU_						
5.	Regression ^a,b^	13.792	3	4.597	465.494	0.000 ^b^
	Residual	0.257	26	0.010		
	Total	14.048	29			
a. Dependent Variable: PWCR_OECDNonEU_						
b. Predictors: (Constant), PWRELF_OECDNonEU_, PUR_OECDNonEU_, PLAE_OECDNonEU_						
6.	Regression ^a,b^	17.412	3.000	5.804	236.939	0.000 ^b^
	Residual	0.637	26.000	0.024		
	Total	18.049	29.000			
a. Dependent Variable: PWCR_OECDASIA_						
b. Predictors: (Constant), PWRELF_OECDASIA_, PUR_OECDASIA_, PLAE_OECDASIA_						
7.	Regression ^a,b^	0.119	3.000	0.040	2865.476	0.000 ^b^
	Residual	0.000	26.000	0.000		
	Total	0.120	29.000			
a. Dependent Variable: PWCR_OECDOceania_						
b. Predictors: (Constant), PWRELF_OECDOceania_, PUR_OECDOceania_, PLAE_OECDOceania_						
8.	Regression ^a,b^	21.561	3	7.187	3143.947	0.000 ^b^
	Residual	0.059	26	0.002		
	Total	21.621	29			
a. Dependent Variable: PWCR_OECDLatAmerica_						
b. Predictors: (Constant), PWRELF_OECDLatAmerica_, PLAE_OECDLatAmerica_, PUR_OECDLatAmerica_						
9.	Regression ^a,b^	0.166	3.000	0.055	2360.164	0.000 ^b^
	Residual	0.001	26.000	0.000		
	Total	0.166	29.000			
a. Dependent Variable: PWCR_OthEU_						
b. Predictors: (Constant), PWRELF_OthEU_, PUR_OthEU_, PLAE_OthEU_						
10.	Regression ^a,b^	4.634	3.000	1.545	1973.765	0.000 ^b^
	Residual	0.020	26.000	0.001		
	Total	4.654	29.000			
a. Dependent Variable: PWCR_OthEurasia_						
b. Predictors: (Constant), PWRELF_OthEurasia_, PUR_OthEurasia_, PLAE_OthEurasia_						
11.	Regression ^a,b^	4.551	3	1.517	2052.844	0.000 ^b^
	Residual	0.019	26	0.001		
	Total	4.570	29			
a. Dependent Variable: PWCR_MENorthAfrica_						
b. Predictors: (Constant), PWRELF_MENorthAfrica_, PUR_MENorthAfrica_, PLAE_MENorthAfrica_						
12.	Regression ^a,b^	4.755	3.000	1.585	4091.570	0.000 ^b^
	Residual	0.010	26.000	0.000		
	Total	4.765	29.000			
a. Dependent Variable: PWCR_OthAfrica_						
b. Predictors: (Constant), PWRELF_OthAfrica_, PLAE_OthAfrica_, PUR_OthAfrica_						
13.	Regression ^a,b^	34.916	3.000	11.639	5058.977	0.000 ^b^
	Residual	0.060	26.000	0.002		
	Total	34.976	29.000			
a. Dependent Variable: PWCR_NonOECDASIA_						
b. Predictors: (Constant), PWRELF_NonOECDASIA_, PUR_NonOECDASIA_, PLAE_NonOECDASIA_						
14.	Regression ^a,b^	508.152	3	169.384	2227.735	0.000 ^b^
	Residual	1.977	26	0.076		
	Total	510.129	29			
a. Dependent Variable: PWCR_China_						
b. Predictors: (Constant), PWRELF_China_, PLAE_China_, PUR_China_						
15.	Regression ^a,b^	34.885	3.000	11.628	2115.586	0.000 ^b^
	Residual	0.143	26.000	0.005		
	Total	35.028	29.000			
a. Dependent Variable: PWCR_India_						
b. Predictors: (Constant), PWRELF_India_, PLAE_India_, PUR_India_						

**Table 4:** Analysis of the degree of regional disparity on policies to combat plastic water pollution in the EU.

Country (2019)	Share of EU Average Mismanaged Plastic Waste (%)	Share of EU Average Mismanaged Plastic Waste to Ocean (%)	Share of EU Average Mismanaged Plastic Waste per Capita (%)	Std. Deviation
Belgium	19.70%	44.21%	27.41%	10.2%
Bulgaria	26.89%	15.00%	61.65%	19.8%
Croatia	151.36%	813.69%	588.16%	274.9%
Cyprus	7.22%	37.54%	96.66%	37.1%
Denmark	3.36%	23.39%	9.36%	8.4%
Estonia	5.18%	135.77%	62.65%	53.4%
Finland	22.61%	0.00%	65.60%	27.2%
France	239.68%	54.13%	59.06%	86.3%
Germany	437.21%	24.06%	84.01%	182.3%
Greece	38.87%	309.41%	59.57%	122.9%
Ireland	23.08%	0.00%	75.87%	31.8%
Italy	334.78%	102.57%	88.73%	112.9%
Latvia	8.24%	70.80%	69.34%	29.2%
Lithuania	8.95%	38.05%	52.02%	17.9%
Malta	2.23%	0.00%	81.50%	37.9%
Netherlands	131.42%	237.80%	123.36%	52.2%
Poland	121.86%	11.48%	51.62%	45.6%
Portugal	32.94%	111.50%	51.70%	33.5%
Romania	450.02%	61.98%	372.95%	167.7%
Slovakia	14.83%	0.00%	43.62%	18.1%
Slovenia	7.28%	79.38%	56.21%	30.1%
Spain	175.57%	75.43%	60.29%	51.2%
Sweden	36.71%	53.81%	58.70%	9.4%
Std deviation	136.0%	169.2%	123.7%	65.5%

**Table 5:** Proposals for improving public policies in the field of combating water pollution caused by microplastics.

Pollution Factor Plastics Polymer (Bioplastics–Dependent Variable)	Effectiveness of Implemented Control Measures	Public Policy Proposals	Graphical Distribution of Correlations with the Reference Pollutant Factor (Bioplastics) *
Marine coatings	Increased in relation to the reference pollutant factor bioplastics (superunit level of pollution reduction in the correlative assessment in relation to the dependent variable of value 4.255).	Introducing higher quality standards for paints used in the shipping industry in order to protect the environment and reduce water pollution caused by microplastics.	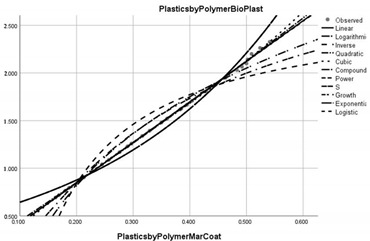
Low-density polyethylene (LDPE) and Linear Low-Density Polyethylene (LLDPE)	Reduced in relation to the reference pollutant factor bioplastics (super-unit level of pollution reduction in the correlative assessment in relation to the dependent variable is close to 0, i.e., 0.043).	Realising an international agreement– on LDPE and LLDPE water pollution whereby plastic manufacturers declare the ingredients in their plastic products and their effects on health.	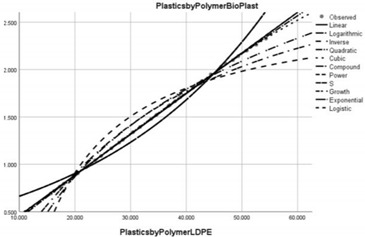
High-density polyethylene (HDPE)	Reduced in relation to the reference pollutant factor bioplastics (super-unit level of pollution reduction in the correlative assessment in relation to the dependent variable is close to 0, i.e., 0.040).	Improving the management of HDPE waste to enable its recycling in terms acceptable to the circular economy.	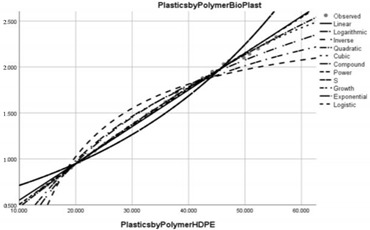
Polypropylene (PP)	Reduced in relation to the reference pollutant factor bioplastics (sub-unit level of pollution re-reduction in the correlative assessment in relation to the dependent variable is close to 0, i.e., 0.032).	New public policies to control the production, consumption and use of PP in areas such as consumer packaging, plastic parts and textiles in the automotive industry.	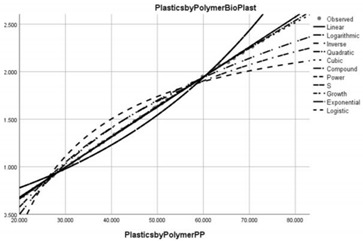
Polystyrene (PS)	Reduced in relation to the reference pollutant factor bioplastics (subunit level of pollution re-reduction in the correlative assessment in relation to the dependent variable is 0.112).	Realising new international agreements that reduce the production and use of PS in the construction, packaging, automotive, pharmaceutical and medical industries.	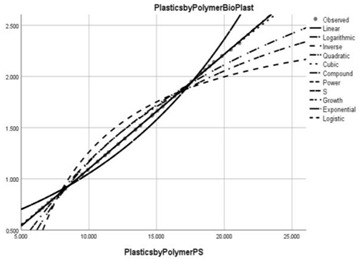
Polyvinyl chloride (PVC)	Reduced in relation to the reference pollutant factor bioplastics (subunit level of pollution re-reduction in the correlative assessment in relation to the dependent variable is 0.046).	Implementation of new measures to improve the collection, treatment and proper disposal of PVC waste used in the construction, textile, electrical, cable and upholstery industries.	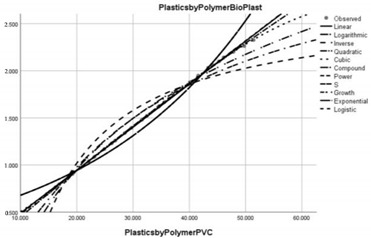
Polyethylene terephthalate (PET)	Reduced in relation to the reference pollutant factor bioplastics (subunit level of pollution re-reduction in the correlative assessment in relation to the dependent variable is 0.089).	Adopting new measures to raise public awareness of the impact of PET on the environment and public health.	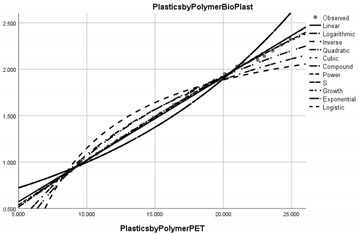
Polyurethane (PUR)	Reduced relative to the reference pollutant factor bioplastics (subunit level of pollution re-reduction in the correlative assessment relative to the dependent variable of 0.130).	New pollution mitigation policies for polyurethane elastomers used for furniture and vehicles as well as sound insulation materials.	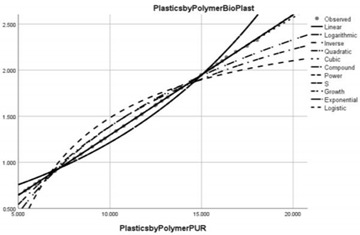
Fibres	Reduced relative to the reference pollutant factor bioplastics (subunit level of pollution re-reduction in the correlative assessment relative to the dependent variable of 0.037).	Adopting new policies to inform the public about the impact of fibres on the environment and public health.	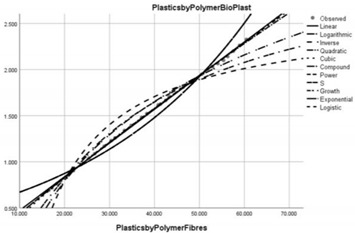
Road marking coatings	Increased in relation to the reference pollutant factor bioplastics (supra-unit level of pollution re-reduction in the correlative assessment in relation to the dependent variable of value 3.441).	Introduction into educational programmes at different levels of information on how to reduce pollution with road markings and pedestrian markings.	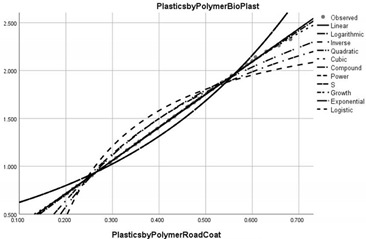
Elastomers (tyres)	Reduced in relation to the reference pollutant factor bioplastics (subunit level of pollution re-reduction in the correlative assessment in relation to the dependent variable of 0.289).	Awareness of the problem of elastomer waste generation in the automotive industry which is significant and has a global environmental impact. Hence the need for sustainable recycling of this waste.	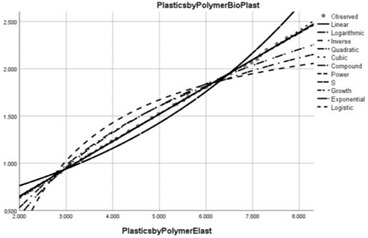

* Arrived at by authors using official statistical data base [55].

## Data Availability

The data presented in this study are available on request from the corresponding author.
